# Mechanisms of *Poria cocos* Wood Colonization: Host Nutrient Depletion and Secondary Metabolite Defense

**DOI:** 10.3390/jof12090666

**Published:** 2026-09-04

**Authors:** Xue Meng, Menghui Han, Xin Gu, Li Zou

**Affiliations:** 1 College of Landscape and Horticulture, Wuhu Vocational and Technical University, Wuhu 241003, China; 101041@whit.edu.cn (X.M.); mx0316@126.com (X.G.); 2 College of Forestry, Northeast Forestry University, Harbin 150040, China; han_mh8128@163.com

**Keywords:** *Poria cocos*, colonization in pine wood, DIA quantitative proteomics, nutritional component analysis, differentially expressed proteins

## Abstract

*Poria cocos* (syn. *Wolfiporia cocos*) is an obligate saprophytic fungus that can produce sclerotia only when cultivated on pine wood. Despite its wide cultivation, the nutritional adaptation of this fungus to pine substrate and the host defensive responses triggered during colonization remain poorly characterized. Elucidating how *Poria cocos* colonizes pine and triggers host defenses is critical to uncover its nutrient dependence. GO and KEGG analyses revealed that protein degradation, carbon metabolism, protein processing in the endoplasmic reticulum, and proteasome pathways were the major enriched pathways, indicating that a large amount of pine wood protein was degraded after *Poria cocos* colonization. Consistent with these omics signatures, the fungus acquired its primary nitrogen source via breakdown of pine structural and metabolic proteins, while pine carbohydrates acted as its main carbon source. Nutritional profiling confirmed the depletion of soluble protein and total sugars in pine wood, alongside elevated defensive polyphenols and flavonoids, reflecting an active stress response from viable pine parenchyma cells upon colonization. No significant shifts in mineral element concentrations were detected in colonized pine wood, indicating that *Poria cocos* selectively absorbs target minerals without altering the overall mineral pool of host wood tissue. Collectively, this work elucidates the core nutritional strategy of *Poria cocos* during pine colonization and identifies key molecular clues to advance optimized artificial cultivation, laying a solid theoretical foundation for revealing its obligate saprophytic lifestyle.

## 1. Introduction

*Poria cocos* (syn. *Wolfiporia cocos*), a polyporaceous fungus whose sclerotia are widely exploited as a valuable medicinal resource [1], faces growing market pressure with annual global demand surpassing 100,000 tons. As an obligate saprophyte, it can only form mature sclerotia when colonizing pine substrates, and current log cultivation consumes 2–3 tons of pine timber to yield 1 ton of fungal sclerotia. This intensive cultivation pattern has caused continuous depletion of pine forest resources and restricted sustainable industrial production [2]. Alongside these industrial constraints, the fundamental biological mechanisms underlying the fungus–pine interaction remain poorly understood. Existing studies have not systematically unraveled the molecular mechanisms governing how *Poria cocos* decomposes pine tissue to acquire carbon and nitrogen nutrients, nor comprehensively characterized the host defensive metabolic responses triggered by fungal colonization. This study aims to dissect the colonization and nutrient utilization strategies of this wood-dwelling saprophyte to tackle these unresolved biological questions.

Cellulose, hemicellulose, and lignin together constitute pine wood [3,4]. *Poria cocos* is classified as a brown-rot fungus. Distinct from most brown-rot fungi that colonize a wide spectrum of wood substrates, *Poria cocos* displays strict host preference and relies exclusively on pine wood to develop mature sclerotia. This obligate dependence on pine substrates represents a key feature that differentiates this species from other brown rot taxa [5,6]. To uncover the molecular processes underlying its wood colonization behavior, proteomics technologies are key tools for studying the mechanisms of fungal wood degradation [7]. Among proteomic strategies, data-independent acquisition (DIA) represents a next-generation analytical workflow. Thanks to its outstanding reproducibility and reliable quantitative accuracy, DIA enables comprehensive and consistent profiling of proteomic changes during the dynamic fungus–pine interaction, making it well-suited to characterize the subtle molecular events underlying *Poria cocos* colonization of pine wood [8]. Nutritional composition analysis is commonly used for detecting nutrients in fruits [9,10], cereals [11,12], timber [13], and other plant materials and is a widely accepted analytical method. This study integrates DIA quantitative proteomics and nutritional composition analysis to characterize dynamic changes in pine wood proteins and nutritional profiles during *Poria cocos* colonization, and explore the patterns by which the fungus utilizes pine substrates, so as to provide references for the development of artificial cultivation media for this fungus.

## 2. Materials and Methods

### 2.1. Materials

Two groups of samples were used in this study: uninoculated *Pinus massoniana* L. wood and *Poria cocos* -colonized *Pinus massoniana* wood containing fungal mycelium. Three biological replicates were set for each group (Q1/Q2/Q3 for uninoculated samples; H1/H2/H3 for colonized samples).

Wood samples were collected from 20-year-old *Pinus massoniana* trees in Yuexi County, Anqing, Anhui Province, China, on 2 March 2026 under overcast conditions (4–12 °C, north wind Level 3, relative humidity 78%). Tissues were taken from the xylem of main branches. Specimens with uniform dimensions (length × diameter: 5 cm × 2 cm) were selected via simple random sampling. The number of biological replicates followed standard protocols for microbial proteomics.

Colonized samples were prepared by inoculating wood substrates with *Poria cocos* strain 5.78 isolated from fungal sclerotia. Inoculated substrates were incubated at 24–28 °C with 60–65% soil water content for 10 months. Samples were confirmed as successfully colonized when the wood surface turned brown, without contamination, and achieved 100% mycelial coverage.

### 2.2. Construction of the Pinus massonian Protein Database

Available public protein resources for *Pinus massoniana* are inadequately annotated, resulting in poor matching efficiency for wood-derived proteins. To overcome this limitation, a protein database of *Pinus massoniana* was built based on transcriptomic sequencing data. DIA-based proteomic analysis combined with nutritional composition analysis was used to examine changes in proteins and nutritional components in pine wood before and after colonization by *Poria cocos*.

#### 2.2.1. Sequencing and Quality Control

Total RNA was extracted from *Pinus massoniana* wood before and after *Poria cocos* colonization using the TRIzol method (Solarbio Life Sciences, Beijing, China) [14]. mRNA was isolated from total RNA using oligo(dT)-based extraction. The purified mRNA was randomly fragmented using divalent cations, and fragmented RNA served as the template for first-strand synthesis using random oligonucleotide primers to generate double-stranded cDNA. The purified cDNA was subjected to end repair, A-tailing, and sequencing adapter ligation. The ligation products were purified and size-selected to obtain cDNA fragments of the desired length, followed by PCR amplification and further purification to construct the final sequencing library. After quality inspection, qualified libraries were pooled based on effective concentration and required sequencing depth and sequenced using Illumina paired-end sequencing (Illumina Inc., San Diego, CA, USA). Raw sequencing data were assessed using FastQC software v0.11.9 (Babraham Institute, Cambridge, UK) [15], and quality trimming was performed using fastp v0.23.2 (open-source; Chen et al., 2018; available at https://github.com/OpenGene/fastp, accessed on 6 March 2026) to ensure data accuracy [16].

#### 2.2.2. Transcriptome Assembly and Gene Annotation

The valid data of the samples were assembled using Trinity v2.8.5 (Broad Institute, Cambridge, MA, USA) for de novo assembly, and the assembled transcript information was statistically analyzed [17].

The transcripts were compared with the CDD, PFAM, and NR databases using the BLAST tool v2.13.0) of the National Center for Biotechnology Information (NCBI, Bethesda, MA, USA), and protein functional annotation information was obtained [18,19].

KEGG annotation information of the transcripts was obtained using the KAAS web server (Kanehisa Laboratories, Kyoto, Japan) [20].

#### 2.2.3. Construction of the In-House Database

Coding sequences of unigenes and transcripts were extracted to construct a *Pinus massoniana* protein database.

### 2.3. DIA Quantitative Proteomics

#### 2.3.1. Protein Extraction

The samples stored at −80 °C were ground into powder with liquid nitrogen, and an appropriate amount of powder was added to lysis buffer (The detailed composition of the lysis buffer is provided in Table S1, followed by cell disruption using ultrasonic assistance (Scientz Biotechnology Co., Ltd., Ningbo, China). Then, an equal volume of Tris-saturated phenol reagent (Solarbio Life Sciences, Beijing, China) was added and stirred at room temperature for 5 min. After thorough mixing, the mixture was centrifuged at room temperature for 5 min to separate the phenol and aqueous phases (Eppendorf AG, Hamburg, Germany), and the upper phenol phase was collected. Four volumes of 0.1 M ammonium acetate in methanol (Sinopharm Chemical Reagent Co., Ltd., Beijing, China) were added to the phenol phase to precipitate proteins overnight. After centrifugation, the precipitate was washed twice with 0.1 M ammonium acetate in methanol and acetone (Sinopharm Chemical Reagent Co., Ltd., Beijing, China), respectively. The precipitate was then redissolved in 8 M urea solution (Solarbio Life Sciences, Beijing, China), and the supernatant protein solution was obtained by centrifugation. Protein concentration was determined using a bicinchoninic acid (BCA kit, Solarbio Life Sciences, Beijing, China) protein assay kit [21].

#### 2.3.2. Protein Digestion and Desalting

Urea was added to a final concentration of 8 M in 100 μg of protein solution (to a total volume equivalent to 200 μg). Then, 5 mM dithiothreitol (DTT, Solarbio Life Sciences, Beijing, China) was added for reduction at 37 °C for 45 min in a thermostatic water bath (HWS series thermostatic water bath, Yiheng Scientific Instrument Co., Ltd., Shanghai, China). Subsequently, 11 mM iodoacetamide (IAA, Solarbio Life Sciences, Beijing, China) was added for alkylation in the dark for 15 min. Finally, 800 μL of 25 mM ammonium bicarbonate solution (Sinopharm Chemical Reagent Co., Ltd., Beijing, China) and 2 μL of trypsin (Promega Corporation, Madison, WI, USA) were added for overnight digestion at 37 °C [22]. The resulting peptides were adjusted to pH 2–3 using 20% TFA (Sigma-Aldrich GmbH, Darmstadt, Germany) and desalted using C18 (Millipore Corporation, Billerica, MA, USA) cartridges. Peptide concentration was finally determined using Thermo Fisher standards and the Pierce™ Quantitative Fluorometric Peptide Assay (Thermo Fisher Scientific Inc., Waltham, MA, USA).

#### 2.3.3. Nanoliter Liquid Chromatography Analysis

Samples were separated using a Vanquish Neo UHPLC nanoflow liquid chromatography system (Thermo Fisher Scientific Inc., Waltham, MA, USA). Mobile phases A and B were 0.1% formic acid (Sigma-Aldrich GmbH, Darmstadt, Germany) in water and 0.1% formic acid in acetonitrile (Sigma-Aldrich GmbH, Darmstadt, Germany), respectively. The injection mode was a trap–elute dual-column system, where the trap column was a PepMap Neo Trap Cartridge (300 μm × 5 mm, 5 μm) and the analytical column was an Easy-Spray™ PepMap™ Neo UHPLC column (150 μm × 15 cm, 2 μm) (Thermo Fisher Scientific Inc., Waltham, MA, USA). The column temperature was maintained at 55 °C using an integrated column oven. The total runtime was 14 min with an effective gradient of 13 min. The sample load was 200 ng, and the flow rate was 2.5 μL/min.

#### 2.3.4. Orbitrap Astral Mass Spectrometry Analysis

Separation was performed using a nanoflow Vanquish Neo chromatography system (Thermo Fisher Scientific Inc., Waltham, MA, USA), and analysis was performed using a Thermo Scientific mass spectrometry system (Thermo Fisher Scientific Inc., Waltham, MA, USA) [23]. The scan range was 380–980 m/z in positive ion mode for precursor ions. The MS1 resolution was 240,000 at 200 m/z. The normalized AGC target was 500%, and the maximum injection time (IT) was 5 ms. MS2 was acquired in DIA mode with 299 scan windows. The isolation window was 2 Th, the HCD collision energy was 25%, the normalized AGC target was 500%, and the maximum IT was 3 ms.

#### 2.3.5. Mass Spectrometry Data Analysis and Protein Quantification

DIA-NN software v1.8.1 (Demichev Lab, Berlin, Germany) was used for DIA mass spectrometry data analysis [24]. The library-free search mode was adopted. The specific parameters included a self-built database, deep-learning-based spectral prediction, sequence-to-spectrum matching, and reanalysis of DIA data to obtain protein identification and quantification results. Both precursor- and protein-level identifications were filtered at a 1% false discovery rate (FDR). The processed data were subjected to bioinformatics analysis.

Protein quantification was performed using the MaxLFQ (v1.6.10) (Max Planck Institute of Biochemistry, Martinsried, Germany) algorithm [25]. The raw intensity of each protein in each sample was normalized by dividing it by the median intensity of all proteins in that sample to obtain relative quantitative values.

#### 2.3.6. Screening of Differentially Expressed Proteins (DEPs)

Student’s *t*-test was used to compare protein abundance between the two groups, and the Benjamini–Hochberg method was applied to correct *p*-values for multiple testing. Differentially abundant proteins were screened based on the thresholds: fold change (FC) ≥ 2.0 or ≤0.5 (|log_2_FC| ≥ 1) and adjusted *p*-value ≤ 0.01.

### 2.4. Determination of Nutritional Components

#### 2.4.1. Soluble Protein

Soluble protein was quantified by Coomassie brilliant blue G-250 (Solarbio Life Sciences, Beijing, China) colorimetry. Briefly, BSA (Solarbio Life Sciences, Beijing, China) gradient solutions were prepared to generate a standard curve at 595 nm. Samples were extracted with distilled water, centrifuged, and the supernatant was subjected to color reaction and absorbance detection to calculate soluble protein content [26].

#### 2.4.2. Total Free Amino Acids

Total free amino acids were quantified via the ninhydrin colorimetric method (Solarbio Life Sciences, Beijing, China). Briefly, leucin standard (Sinopharm Chemical Reagent Co., Ltd., Beijing, China) solutions were used to plot a standard curve at 580 nm; samples were extracted with 10% acetic acid (Sinopharm Chemical Reagent Co., Beijing, China), diluted with acetate buffer, and heated with ninhydrin reagent for color development before absorbance measurement [27].

#### 2.4.3. Total Polyphenols

Total polyphenols were determined via the Folin–Ciocalteu (Solarbio Life Sciences, Beijing, China) colorimetric method. Gallic acid (Sinopharm Chemical Reagent Co., Ltd., Beijing, China) was used to create a standard curve at 765 nm. Samples were extracted in boiling water, mixed with Folin reagent and 7.5% sodium carbonate (Sinopharm Chemical Reagent Co., Ltd., Beijing, China) solution, and subjected to color development before absorbance measurement [28].

#### 2.4.4. Total Sugar

Total sugar was determined by acid hydrolysis phenol-sulfuric acid colorimetry according to GB/T 15672-2009. Briefly, samples were hydrolyzed with hydrochloric acid (Sinopharm Chemical Reagent Co., Ltd., Beijing, China) in a boiling water bath; glucose standard (Sinopharm Chemical Reagent Co., Ltd., Beijing, China) solutions were used to draw a standard curve at 490 nm, and absorbance of treated sample solutions was measured for quantification [29].

#### 2.4.5. Total Flavonoids

Total flavonoids were determined by aluminum nitrate (Sinopharm Chemical Reagent Co., Ltd., Beijing, China) colorimetry with rutin as standard (Sinopharm Chemical Reagent Co., Ltd., Beijing, China) at 510 nm. Samples were extracted with 60% ethanol (Sinopharm Chemical Reagent Co., Ltd., Beijing, China) in a water bath, followed by successive addition of sodium nitrite, aluminum nitrate and sodium hydroxide (Sinopharm Chemical Reagent Co., Ltd., Beijing, China) solutions for color development before absorbance detection [30].

#### 2.4.6. Crude Fat

Crude fat was determined by Soxhlet extraction (SXT-06 Soxhlet extractor, Shanghai Peiou Analytical Instrument Co., Ltd., Shanghai, China) following GB 5009.6-2016. Samples were extracted with petroleum ether (Sinopharm Chemical Reagent Co., Ltd., Beijing, China) under a constant-temperature water bath; after extraction, the solvent was evaporated, and the residue was dried to constant weight to calculate fat content [31].

#### 2.4.7. Mineral Elements (P, K, Ca, and Mg)

Mineral elements including P, K, Ca and Mg were determined by inductively coupled plasma-mass spectrometry (ICP-MS, Thermo Fisher Scientific Inc., Waltham, MA, USA) according to GB 5009.268-2016. Briefly, dried samples were weighed and soaked with nitric acid (Sinopharm Chemical Reagent Co., Ltd., Beijing, China) in polytetrafluoroethylene digestion tanks, followed by gradient heating digestion in an oven. After digestion and natural cooling, the solution was heated to remove excess acid, then a constant volume was prepared with diluted nitric acid, and a blank control was set simultaneously. Element concentrations were quantified by the internal standard method through ICP-MS detection [32].

#### 2.4.8. Total Nitrogen

Total nitrogen was determined via the Kjeldahl method (NY/T 2017-2011). Briefly, samples were digested with concentrated sulfuric acid (Sinopharm Chemical Reagent Co., Ltd., Beijing, China) and a digestion catalyst (Sinopharm Chemical Reagent Co., Ltd., Beijing, China) in an SKD-20S2 digestion furnace (Peiou Analytical Instrument Co., Ltd., Shanghai, China) at high temperature until the solution turned uniform brown-black. After cooling, the digested liquid was alkalized and distilled; the released ammonia was absorbed by boric acid (Sinopharm Chemical Reagent Co., Ltd., Beijing, China) solution, then titrated with standard sulfuric acid using methyl red-bromocresol green mixed indicator (Sinopharm Chemical Reagent Co., Ltd., Beijing, China) on an SKD-1100 automatic Kjeldahl nitrogen analyzer (Peiou Analytical Instrument Co., Ltd., Shanghai, China) to calculate total nitrogen content [33].

### 2.5. Bioinformatics Analysis

GO functional enrichment and KEGG pathway enrichment analyses were performed using the ClusterProfiler package (v4.4.4, R 4.2.0) (R Core Team, Vienna, Austria) (*p*-value ≤ 0.05) [34].

### 2.6. Data Statistics and Analysis

Three independent biological replicates were established for each treatment group, with each replicate derived from distinct batches of pine wood material to ensure sample independence. Sample independence was guaranteed by experimental design. Owing to the limited sample size (n = 3), formal tests for normality and homogeneity of variance have low statistical power; hence, these assumptions were adopted for Student’s *t*-test. Results are presented as mean ± standard deviation. Student’s *t*-test was performed using SPSS 22.0. (IBM Corp., Armonk, NY, USA) Differences with *p* < 0.05 were considered statistically significant.

## 3. Results

### 3.1. Self-Built Transcriptome Database of Pine Wood

Transcriptome sequencing of *Pinus massoniana* samples generated 21 Gb raw data containing 140,188,086 raw reads, with a Q30 ratio of 96.12%, demonstrating high sequencing reliability. De novo assembly using Trinity software produced 362,835 transcripts and 257,445 unigenes. For the assembled unigenes, the N50 length reached 805 bp, N90 was 238 bp, the average sequence length was 553.16 bp, the maximum sequence length was 12,071 bp, and the total assembled length was 142,408,966 bp. In functional annotation analysis, 153,682 unigenes obtained homologous matches in the NR database, accounting for 59.70% of all 257,445 unigenes; a total of 168,396 unigenes were annotated in at least one of seven public databases (NR, NT, CDD, PFAM, GO, KEGG, Swiss-Prot), occupying 65.41% of the total unigene set. Correspondingly, 34.59% of unigenes (89,049 sequences) lacked identifiable homologous sequences across all tested databases, which may represent species-specific novel coding sequences, non-coding transcripts, or highly divergent genes with insufficient homologous entries in current public databases. Assembly metrics including unigene N50, average length and overall annotation ratio were comparable to previously published de novo transcriptome datasets of congeneric species such as *Pinus sylvestris* and *Pinus radiata* [35,36], confirming acceptable assembly continuity and adequate sequencing depth for subsequent proteomic database construction. Based on these annotated unigene sequences, a customized *Pinus massoniana* protein database containing 140,347 predicted protein sequences was constructed for subsequent proteomic matching and identification.

### 3.2. DIA Quantitative Proteomics Results

#### 3.2.1. Protein Identification and Quality Control

By comparing against the 140,347 protein sequences in the *Pinus massoniana* self-built database, 4298 peptides and 2444 proteins were identified, and a total of 2396 proteins were ultimately quantified. Quality control analysis of the identified proteins showed that, according to the distribution of missed cleavage sites, peptides with 0 missed cleavage sites accounted for 69.7%, those with 1 missed cleavage site accounted for 24.8%, and those with 2 or more missed cleavage sites accounted for 5.5%, indicating that enzymatic digestion was complete. The identification results are shown in Table 1.

**Figure 1:**
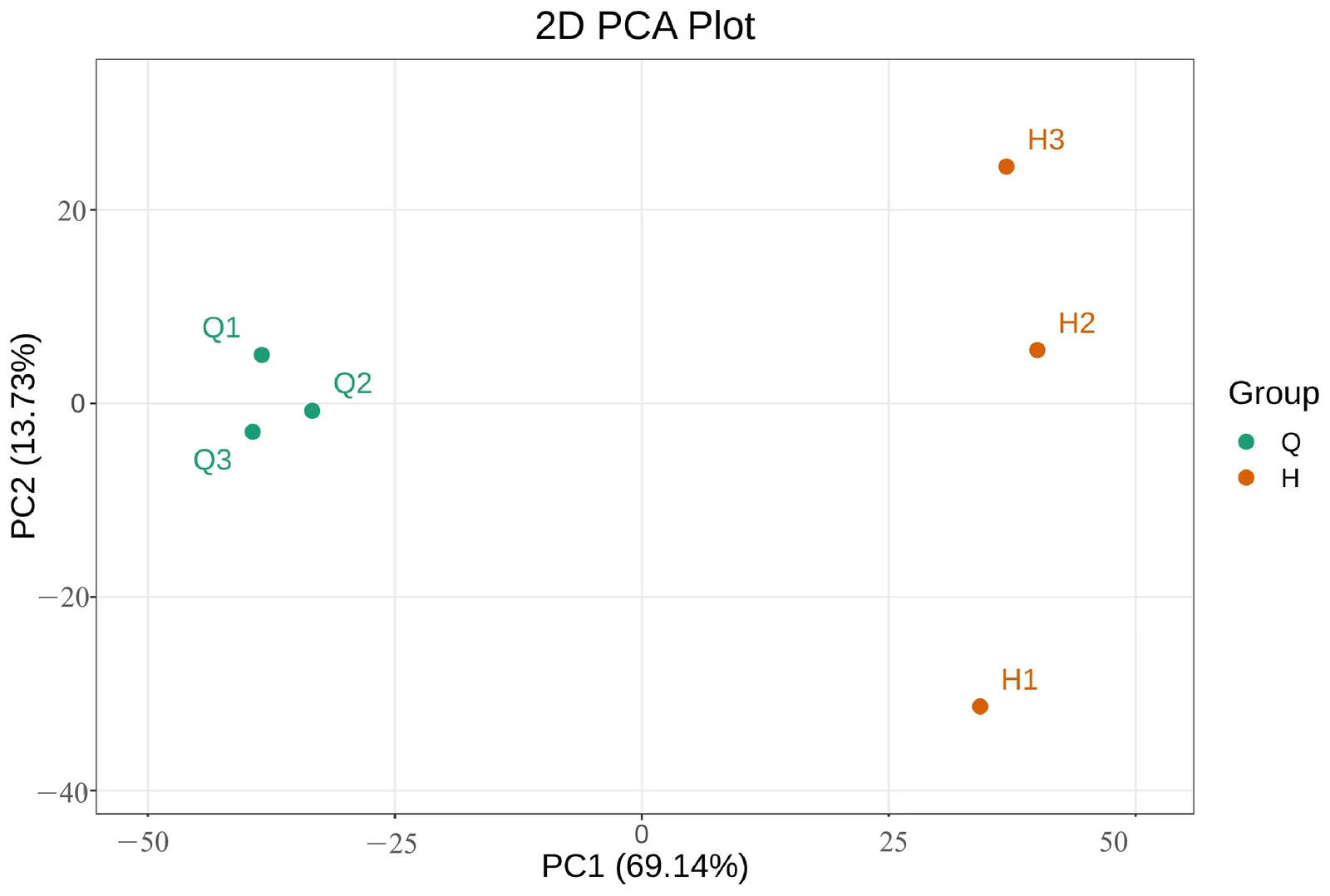
PCA results. PC1 and PC2 represent the first and second principal components, respectively; the contribution of each component is expressed as a percentage. Each dot represents a sample, and the yellow and green dots represent different samples.

**Figure 2:**
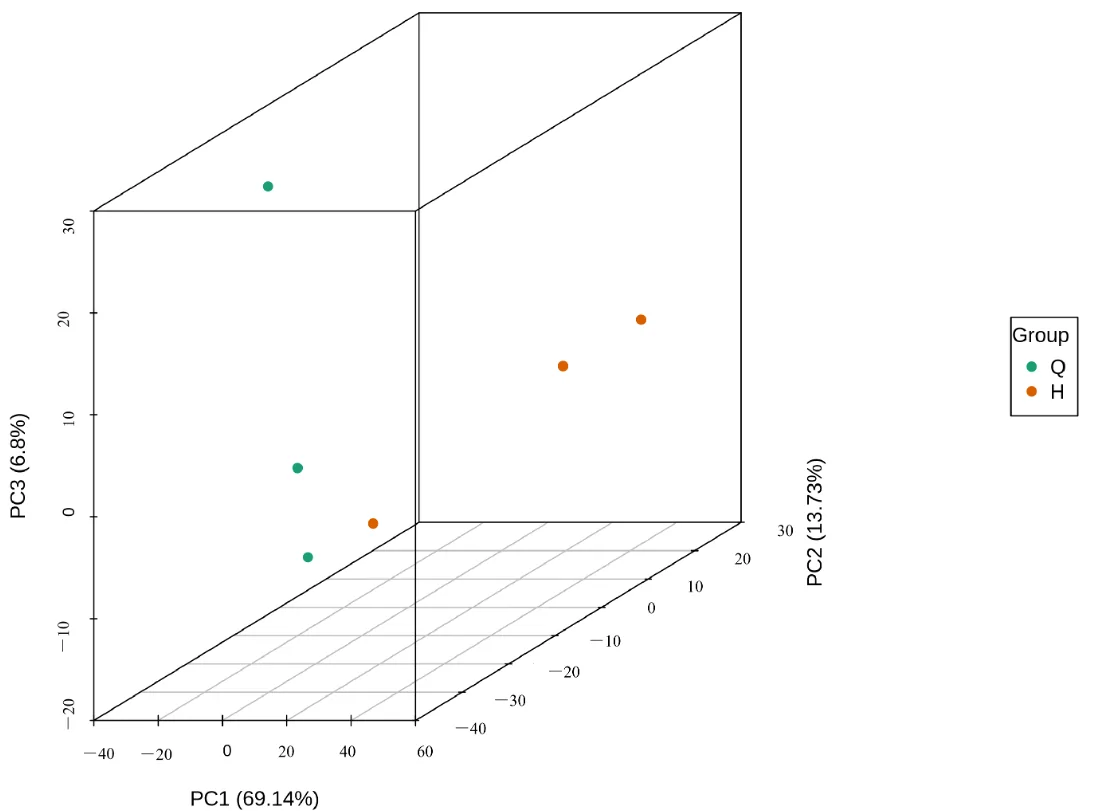
3D PCA results. PC1, PC2, and PC3 represent the first, second, and third principal components, respectively; each dot represents a sample, and the yellow and green dots represent different samples.

PCA revealed that uncolonized pine wood (Group Q) and pine wood colonized by *Poria cocos* (Group H) were clearly separated in the PC space (Figure 1). Group Q clustered on the negative side of PC1, whereas Group H clustered on the positive side of PC1, indicating significant changes in the pine wood proteome after *Poria cocos* colonization. The Pearson correlation coefficients among samples within the same group were all >0.9, while those between groups were significantly reduced, confirming the reliability of the data. The three-dimensional PCA plot further confirmed good clustering of the biological replicates within each group, with no overlap observed between the two groups (Figure 2). These results further reveal the high reproducibility and reliability of the experimental data. The differential protein abundances indicated that colonization by *Poria cocos* induced broad proteomic changes in pine wood, which may further disturb related physiological and metabolic pathways.

#### 3.2.2. DEPs

Based on the criteria for significant differential expression (FC ≥ 2.0 or FC ≤ 0.5 and *p*-value ≤ 0.01), changes in pine wood proteins before and after *Poria cocos* colonization were analyzed. A total of 784 DEPs were identified, accounting for 32.72% of all quantified proteins; 595 were downregulated proteins (24.83%) and 189 were upregulated proteins (7.89%). The statistics of differential expression analysis results are shown in Table 2.

**Figure 3:**
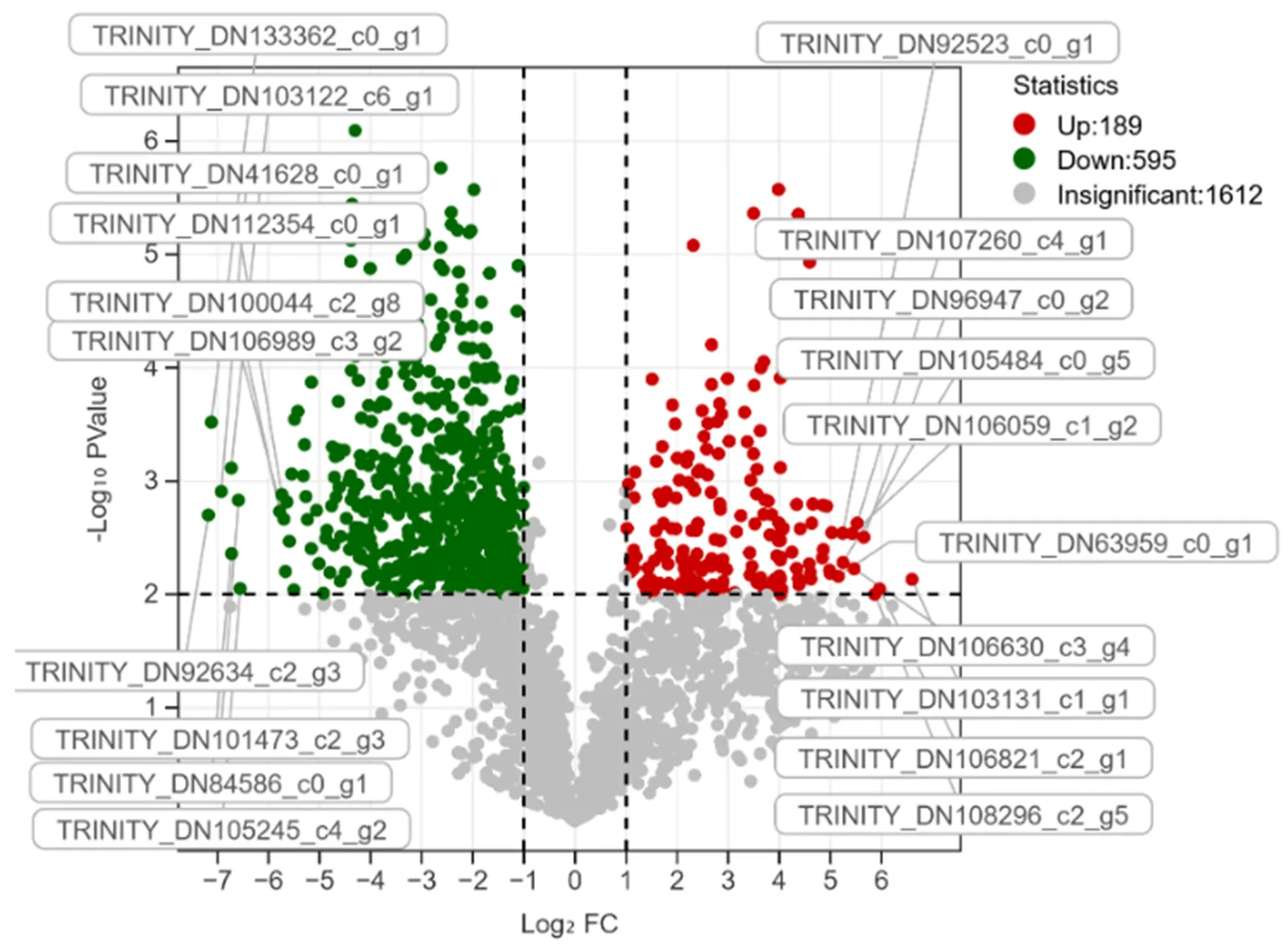
Volcano plot of DEPs. The horizontal axis represents the log2 fold change, and the vertical axis represents the log10 *p*-value. Red dots represent upregulated proteins, and green dots represent downregulated proteins.

As shown in Figure 3, The most significantly upregulated protein TRINITY_DN103131_c1_g1 (predicted mitochondrial regulatory protein) participated in multiple cellular metabolic and stress response pathways; the second most induced protein TRINITY_DN106821_c2_g1 (predicted ribosomal protein) was involved in ribosome biogenesis and cytoplasmic translation processes, and the third most induced protein TRINITY_DN108296_c2_g5 (predicted translation-related GTP-binding protein) functioned in eukaryotic translation and ribosomal assembly pathways.

Meanwhile, the most significantly downregulated protein TRINITY_DN92634_c2_g3 (predicted glycolytic enzyme) was central to glycolysis and carbohydrate energy metabolism pathways; the second most repressed protein TRINITY_DN133362_c0_g1 (predicted cytoplasmic chaperone protein) participated in protein folding and intracellular metabolic homeostasis, and the third most repressed protein TRINITY_DN41628_c0_g1 (predicted oxidoreductase) was involved in general cellular redox and secondary metabolic processes.

**Figure 4:**
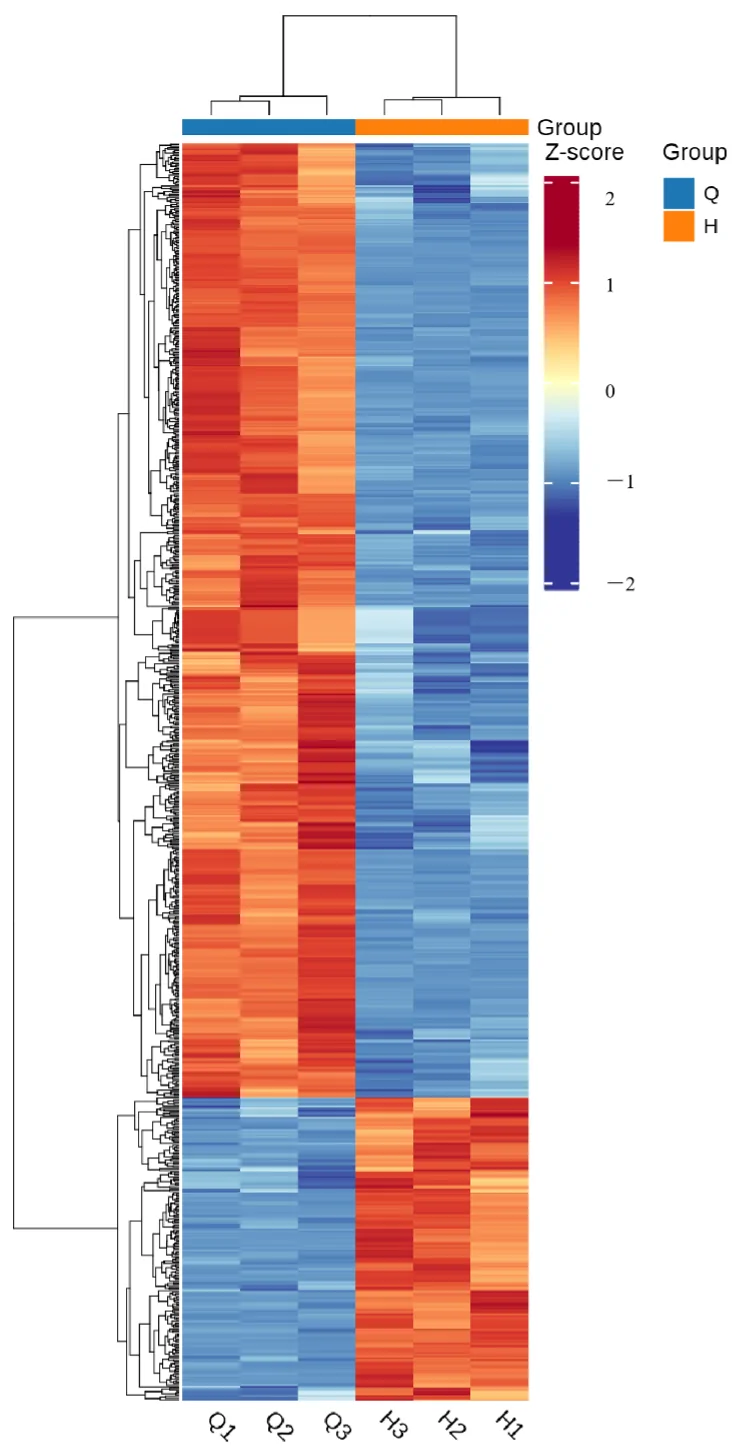
Cluster heatmap of all DEPs. Rows represent proteins, and columns represent samples. Shorter clustering branches indicate higher similarity. Red indicates high expression, and blue indicates low expression.

Cluster analysis of the 784 DEPs was performed, and the results are shown in Figure 4. All Q and H samples were grouped into two clusters, indicating significant differences in protein expression before and after *Poria cocos* colonization. The three biological replicates within each group (Q1/Q2/Q3 and H1/H2/H3) clustered together, indicating good reproducibility within groups. In the figure, red represents high-expression proteins and blue represents low-expression proteins. In the H group, the blue region corresponds to 595 downregulated proteins after *Poria cocos* colonization in pine wood, while the red region corresponds to 189 upregulated proteins, consistent with the differential protein screening results.

#### 3.2.3. GO-Based Functional Annotation and Enrichment Analysis of DEPs

**Figure 5:**
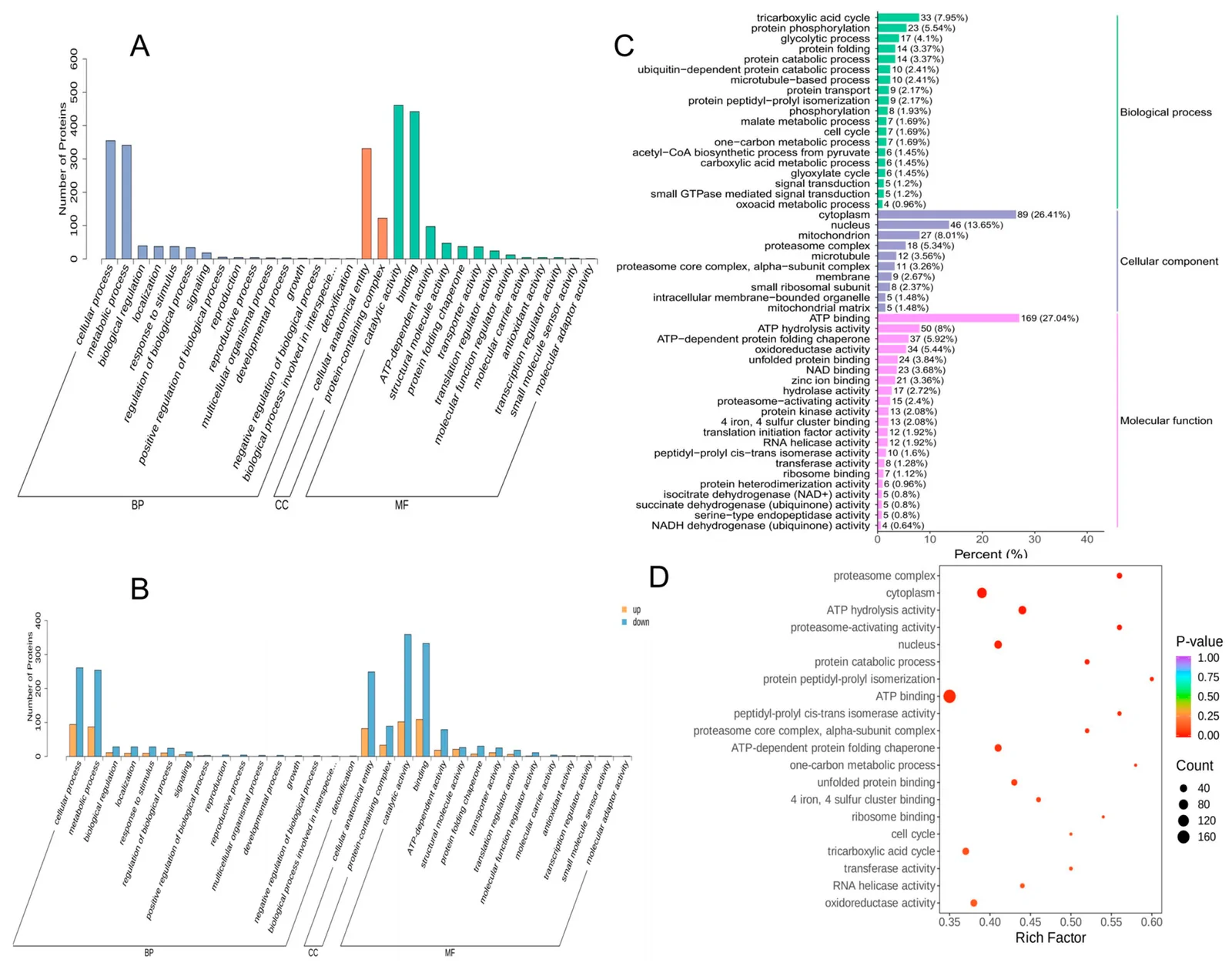
GO functional annotation and enrichment analysis. A bar chart of GO-annotated DEPs is shown in Panel (**A**). The horizontal axis represents second-level GO terms, and the vertical axis represents the number of DEPs in each GO term. Bars in different colors indicate different primary GO classifications. A bar chart of up- and downregulated DEPs is shown in Panel (**B**), where upregulated proteins are shown in yellow and downregulated proteins in blue. A bar chart of GO enrichment analysis of DEPs is shown in Panel (**C**). The horizontal axis represents the proportion of DEPs annotated to each GO term, and the y-axis represents GO term names. The numbers indicate the number of DEPs annotated to each term. The values in parentheses represent the ratio of the number of annotated DEPs to the total number of annotated DEPs. The labels on the rightmost side indicate the primary GO classifications. A bubble plot of GO enrichment analysis of DEPs is shown in Panel (**D**). The horizontal axis represents the enrichment factor; a higher enrichment factor indicates a higher degree of enrichment. The vertical axis represents GO term names. Dot color ranges from blue to red, indicating decreasing *p*-values; smaller *p*-values indicate higher statistical significance. The dot size represents the number of DEPs annotated to each term.

GO functional analysis (Figure 5A) revealed that most proteins were enriched in biological processes such as cellular processes, metabolic processes, biological regulation, stress response, and signal transduction. These basic process categories reflect the overall disturbance of fundamental physiological activities in pine wood triggered by fungal colonization. In terms of cellular components, many protein complexes and structural proteins were affected. The alteration of structural protein assemblies directly impairs the normal cell wall and intracellular structural stability of pine tissues. In terms of molecular function, the DEPs were associated mainly with catalytic activity and binding activity. Catalytic and binding functions underpin nearly all enzymatic and molecular interaction reactions of host cells. As shown in Figure 5B, in the functional annotation of up- and downregulated DEPs, the number of downregulated proteins in the core biological process terms “cellular process” and “metabolic process” was much higher than that of upregulated proteins, indicating that the basal cellular and metabolic activities of pine wood were suppressed after *Poria cocos* colonization. However, upregulated proteins were also observed in pathways such as stress response, which may reflect the activation of defense-related secondary metabolism in pine wood in response to colonization. In the cellular component category, including “cellular anatomical entity” and “protein-containing complex,” downregulated proteins were far more abundant than upregulated proteins, indicating that *Poria cocos* colonization significantly affected a large number of structural proteins and protein complexes in pine wood, with an overall downregulated trend. This massive reduction in structural proteins weakens the physical defense barrier of pine xylem against fungal invasion. In molecular function enrichment, downregulated proteins were also dominant in catalytic activity and binding activity, suggesting that enzyme synthesis and basic metabolic activities were inhibited during *Poria cocos* colonization of pine wood. The presence of upregulated proteins related to protein folding chaperones, structural protein activity, and transporter protein activity may be attributed both to host protein degradation and to fungal hyphal growth and metabolism within pine wood. The induced chaperone proteins also represent an endogenous repair attempt of pine cells to alleviate protein damage caused by the fungus. In the GO enrichment bar plot of DEPs (Figure 5C), the major biological process terms included the tricarboxylic acid (TCA) cycle, protein phosphorylation, glycolysis, and protein folding. In the cellular component category, the main terms were cytoplasm, nucleus, mitochondria, and proteasome complex, indicating that the ubiquitin–proteasome system plays an important role during *Poria cocos* colonization of pine wood. In molecular function, the core terms included ATP binding, ATP hydrolytic activity, and ATP-dependent protein folding chaperones. The bubble plot (Figure 5D) shows significant enrichment differences in ATP binding, proteasome complex, protein catabolic process, and ATP hydrolysis activity. The ATP binding term had the largest number of proteins, indicating that the highest number of DEPs was involved in this pathway. The proteasome complex showed the highest enrichment level, suggesting that this function represents the most prominent biological process during *Poria cocos* colonization of pine wood. Overall, these results indicate that *Poria cocos* colonization leads to significant alterations in protein degradation and energy metabolism pathways in pine wood.

#### 3.2.4. KEGG Functional Annotation and Enrichment Analysis of DEPs

**Figure 6:**
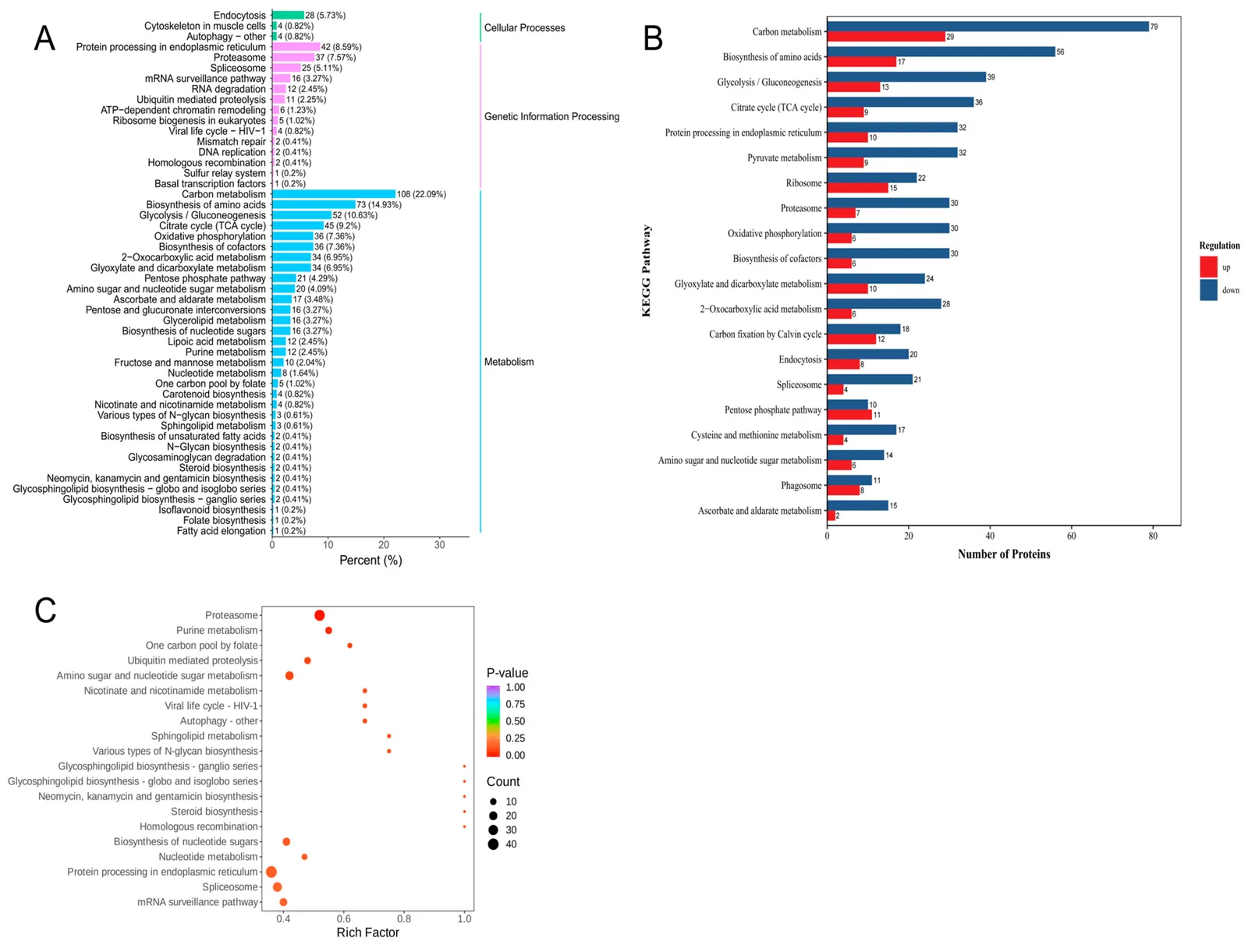
KEGG functional annotation and enrichment analysis. The KEGG classification bar chart of DEPs is shown in Panel (**A**). The horizontal axis represents the ratio of the number of DEPs annotated to a given pathway to the total number of annotated proteins. The vertical axis represents KEGG pathway names, and the numbers indicate the number of DEPs annotated to each pathway. The value in parentheses represents the ratio of the number of DEPs annotated to the pathway to the total number of annotated proteins. The labels on the right indicate the primary KEGG pathway categories. A comparative bar chart of KEGG classification for upregulated and downregulated DEPs is shown in Panel (**B**). The horizontal axis represents the number of DEPs annotated to each functional category, and the vertical axis represents KEGG functional category names. Red bars indicate upregulated DEPs, and blue bars indicate downregulated DEPs. Only the top 20 KEGG pathways based on annotation ranking are shown in this panel. A bubble plot of KEGG enrichment analysis of DEPs is shown in Panel (**C**). The horizontal axis represents the enrichment factor; a higher enrichment factor indicates a higher degree of enrichment of DEPs. The vertical axis represents KEGG pathways. The dot color gradient from blue to red indicates decreasing *p*-value; the smaller the *p*-value, the higher the statistical significance, and all terms with *p*-value < 0.05 were defined as significantly enriched GO terms in this analysis. The dot size represents the number of DEPs annotated to the corresponding function.

As shown in Figure 6A, metabolism represented the largest category, with carbon metabolism showing the highest proportion and being the most enriched KEGG pathway among DEPs, followed by amino acid biosynthesis, glycolysis, and TCA cycle pathways. These pathways are closely related to energy metabolism and biosynthesis, which indicates that energy metabolism underwent significant changes during *Poria cocos* colonization of pine wood, thereby providing energy for fungal colonization. In the category of genetic information processing, protein processing in the endoplasmic reticulum and the proteasome pathway accounted for the highest proportion of DEPs, indicating activation of the ubiquitin–proteasome system and protein folding processes; these findings are consistent with the GO analysis results. In the cellular process category, endocytosis showed the highest proportion, which is closely related to intracellular transport and signal transduction, suggesting that *Poria cocos* colonization affects transcriptional regulation in pine wood. As shown in Figure 6B, the metabolic pathways accounted for the largest overall proportion. In carbon metabolism, 79 proteins were downregulated, far exceeding the 29 upregulated proteins, which indicates that the carbon metabolism pathway in pine wood was suppressed by *Poria cocos* colonization. Similarly, amino acid biosynthesis, glycolysis, and the TCA cycle were dominated by downregulated proteins, suggesting that both primary metabolism and biosynthesis in pine wood were inhibited. The presence of upregulated proteins in certain metabolic pathways may be associated with the metabolic activity of *Poria cocos* hyphae within pine wood. In the genetic information processing category, protein processing in the endoplasmic reticulum, proteasome, and ribosome pathways were also composed mainly of downregulated proteins, indicating an overall inhibition of protein synthesis in pine wood after colonization. The upregulated proteins in these pathways may reflect fungal metabolism and stress responses of living pine cells following *Poria cocos* colonization. In the cellular process category, endocytosis and phagosome-related proteins showed slightly more downregulation than upregulation, indicating that cellular transport and defense functions in pine wood were also affected. As shown in Figure 6C, the proteasome pathway occupied the highest position in the plot and showed a large node size, indicating a high degree of enrichment and significant differences, consistent with the GO analysis results. This confirms that the ubiquitin–proteasome system is activated during *Poria cocos* colonization of pine wood. The endoplasmic reticulum protein processing pathway also showed the largest node size, indicating the highest number of enriched proteins and suggesting disruption of protein folding and endoplasmic reticulum quality control processes, with significant enrichment of ubiquitin-mediated proteolysis, further highlighting the central role of the ubiquitin–proteasome system in stress responses. Amino sugar and nucleotide sugar metabolism, purine metabolism, and nicotinate and nicotinamide metabolism pathways were significantly enriched, indicating that energy metabolism and nucleotide metabolism in pine wood were strongly affected after colonization. In the genetic information processing category, pathways such as the spliceosome and mRNA surveillance system indicate that RNA processing and transcriptional regulation in pine wood were affected after *Poria cocos* colonization.

### 3.3. Changes in Nutrient Composition of Pine Wood Before and After Colonization and of Poria cocos Sclerotia

Because *Poria cocos* is a fungal tissue and is not compatible with the pine wood protein database, it is not suitable for direct comparison of protein differences with pine wood. DIA proteomics only detected protein changes in pine wood before and after *Poria cocos* colonization. To investigate the differences in nutritional composition between healthy pine wood, pine wood colonized by *Poria cocos*, and *Poria cocos* sclerotia, the nutritional components of the three samples were determined in this study.

**Figure 7:**
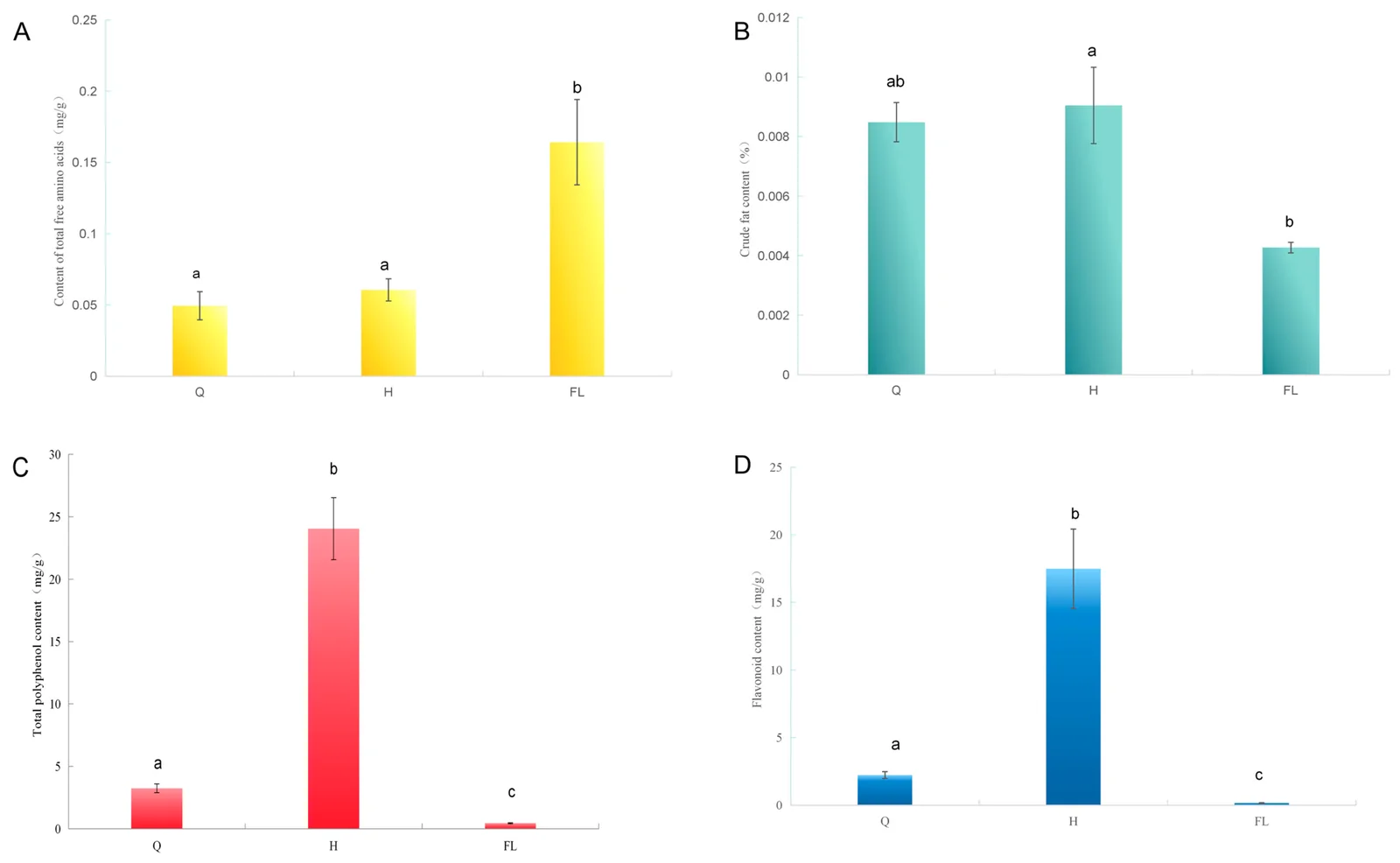

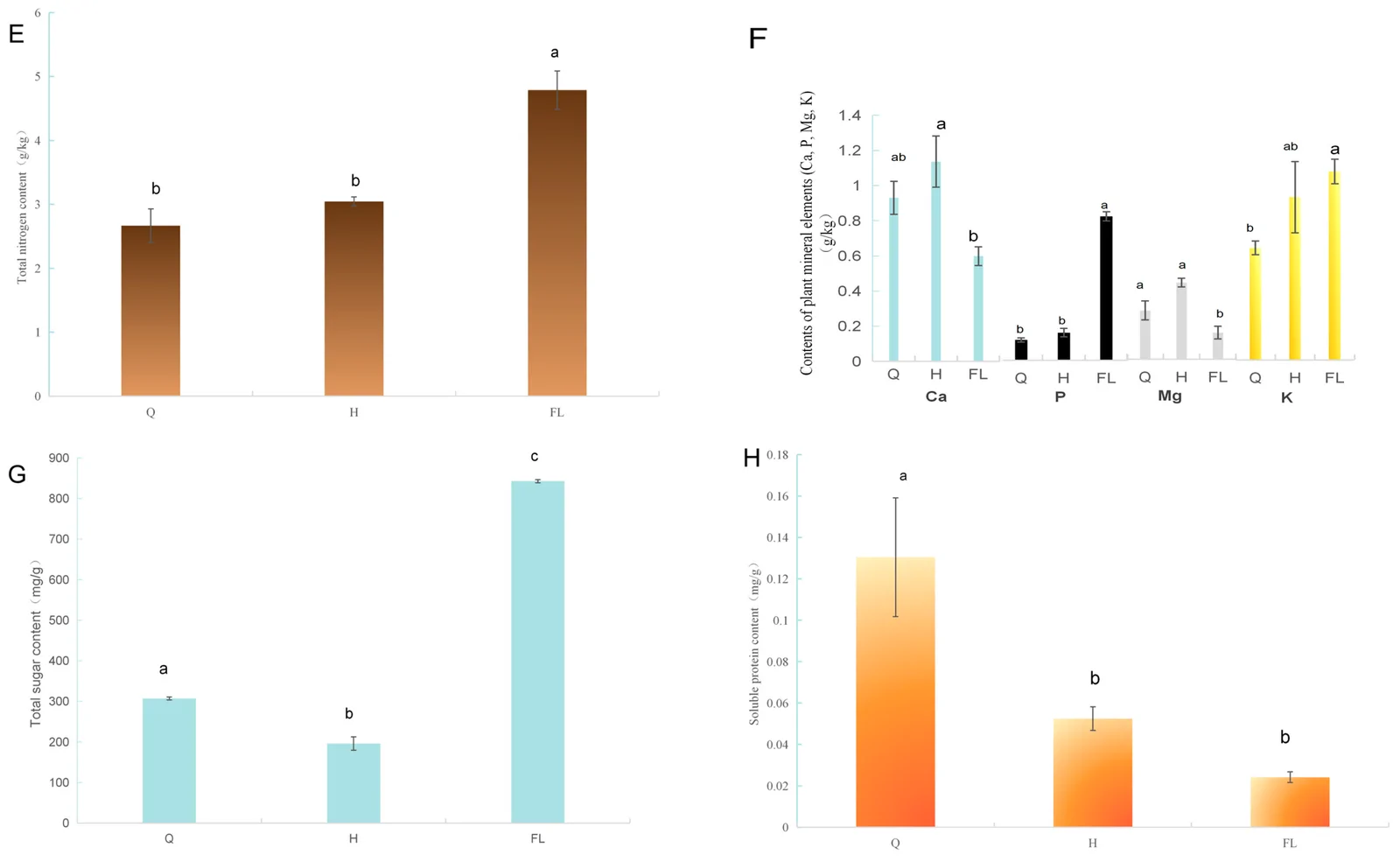
Histogram of nutritional component analysis of pine wood before and after colonization and of *Poria cocos* sclerotia. (**A**) Total free amino acid content; (**B**) crude fat content; (**C**) total polyphenol content; (**D**) total flavonoid content; (**E**) total nitrogen content; (**F**) contents of mineral elements (Ca, P, Mg, K); (**G**) total sugar content; (**H**) soluble protein content. In all panels, the x-axis represents sample groups (Q, uncolonized pine wood; H, *Poria cocos*-colonized pine wood; FL, *Poria cocos* sclerotia), and the y-axis indicates the concentration of corresponding nutritional components. Different lowercase letters above bars denote significant differences between groups (*p* < 0.05).

As shown in Figure 7 and Table 3, soluble protein content changed significantly before and after *Poria cocos* colonization (*p* < 0.05), with a maximum of 0.13 mg/g before colonization and 0.05 mg/g after colonization, representing a 62.01% decrease. The lowest content was observed in *Poria cocos* sclerotia, indicating that soluble proteins in pine wood were extensively consumed during colonization and may serve as a nitrogen source for *Poria cocos*. The total sugar content changed significantly before and after colonization (*p* < 0.01). decreasing from 307.25 mg/g before colonization to 196.07 mg/g after colonization, a reduction of 36.19%. The highest level was observed in *Poria cocos* sclerotia, indicating that sclerotia are the main site of sugar accumulation and that sugars from pine wood are utilized by *Poria cocos* as a carbon source. The total polyphenol content showed a very significant change before and after colonization (*p* < 0.01), increasing from 3.25 mg/g before colonization to 24.05 mg/g after colonization, an increase of 640.38%. The lowest level was detected in *Poria cocos* sclerotia, which indicates that colonization by *Poria cocos* activated defense responses in living cells of pine wood, leading to strong biochemical reactions. Similarly, total flavonoid content also showed extremely significant changes (*p* < 0.01), increasing from 2.22 mg/g to 17.49 mg/g (an increase of 687.09%), with the lowest level in *Poria cocos* sclerotia, suggesting that flavonoid accumulation is also a result of defense activation in the living cells of pine wood.

### 3.4. Correlation Analysis of Nutritional Components and Proteomics

Integrative interpretation linking variations in nutritional components and the functions of DEPs is presented in Table 4. The reduction in soluble protein content may be associated with protein degradation processes in pine wood, potentially driven by proteolytic enzymes. This observation is consistent with the results of GO enrichment analysis. The decline in total sugar content may be associated with the mobilization of wood carbohydrates via proteins involved in glycolysis, starch and sucrose metabolism, which could provide carbon substrates for *Poria cocos*. The elevated levels of total polyphenols and flavonoids after colonization may be associated with the activation of host defensive responses; proteins associated with phenylpropanoid biosynthesis were enriched, which may facilitate the biosynthesis of defensive polyphenols and flavonoids against fungal colonization.

## 4. Discussion

In this work, we combined DIA quantitative proteomic profiling and nutritional component quantification to characterize molecular and nutritional alterations occurring in *Pinus massoniana* wood upon colonization by *Poria cocos*. We detected pronounced depletion of structural and metabolic proteins, alongside reduced carbohydrate concentrations in colonized xylem. This measurable decline in host macromolecules may tentatively suggest that these pine-derived compounds could serve as potential carbon and nitrogen substrates for W. cocos. Additionally, elevated defensive metabolites detected in colonized wood imply that viable pine ray parenchyma cells may trigger stress-associated metabolic shifts following fungal colonization. In this study, a theoretical basis is provided for elucidating the obligate saprophytic mechanism of *Poria cocos*.

### 4.1. Degradation of Large Amounts of Pine Wood Proteins as a Nitrogen Source by Poria cocos

A comparison of protein changes in pine wood before and after *Poria cocos* colonization revealed that 595 proteins were significantly downregulated, and the main enriched biological processes were proteins annotated to protein degradation, transport, and ATPase activity. Nutritional analysis also showed that the soluble protein level in pine wood decreased by 62.01% after colonization. These results are consistent with documented wood degradation mechanisms of brown-rot fungi, which are reported to directly secrete proteases to break down wood proteins as nitrogen substrates for growth [37,38]. GO and KEGG analyses showed enrichment of the ubiquitin–proteasome system and the protein processing in the endoplasmic reticulum pathway, further suggesting that *Poria cocos* promotes protein degradation by inducing protein folding and degradation processes in pine wood [39,40]. Nutritional composition analysis showed no significant change in total free amino acids before and after colonization (*p* > 0.05), which also may be consistent with the rapid uptake of low-molecular-weight free amino acids by *Poria cocos*. In summary, the utilization of pine wood proteins by *Poria cocos* occurs primarily through protein degradation.

### 4.2. Source Analysis of Upregulated Proteins in Pine Wood After Poria cocos Colonization

In the protein analysis experiment, changes in the protein content of pine wood before and after colonization were compared. There were 189 upregulated proteins. These upregulated proteins may originate mainly from two sources. One source is that after *Poria cocos* colonization, fungal hyphae enter the pine wood, and during analysis it is difficult to separate wood from fungal mycelia; therefore, fungal proteins may match homologous proteins in the pine wood database. As a result, some proteins from *Poria* hyphae are misidentified as pine wood proteins. This phenomenon, where fungal proteins and metabolic enzymes are misassigned to the host because of sequence homology in plant–fungus interaction proteomics, has been confirmed by several studies [41]. The other source of upregulated proteins is possibly stress-related defense proteins produced by ray parenchyma cells of pine wood. Some parenchyma cells in pine wood remain alive, and after *Poria cocos* colonization, these living cells may respond to stress by producing proteins related to defense against fungal colonization [42].

### 4.3. Utilization of Total Sugars as the Carbon Source by Poria cocos During Colonization of Pine Wood

Nutrient composition analysis revealed that the total sugar content of pine wood significantly decreased by 36.19% after *Poria cocos* colonization, whereas the total sugar content in *Poria cocos* sclerotia significantly increased, suggesting that *Poria cocos* utilizes mainly pine wood carbohydrates as its carbon source. DIA quantitative proteomics showed that proteins involved in carbon metabolism, glycolysis, and the TCA cycle were significantly downregulated, suggesting reduced abundance of carbon metabolism-related proteins in pine wood. This observation is also consistent with the finding that brown-rot fungi can utilize host cellulose and hemicellulose through extracellular enzyme secretion [43,44]. In this study, the decrease in the total sugar content of pine wood was closely associated with the degradation of carbon metabolism-related proteins, consistent with indirect molecular-level evidence that *Poria cocos* may potentially obtain carbon substrates from pine wood carbohydrates.

### 4.4. Initiation of the Defense Mechanism of Pine Wood Against Poria cocos Colonization via Secretion of Polyphenols and Flavonoids from Living Cells

Most cells in pine wood are dead; however, nutrient analysis showed that after *Poria cocos* colonization, total polyphenols and flavonoids increased by 640.38% and 687.09%, respectively. This suggests that pine wood initiated a defense response, and these compounds were not derived from dead cells. Instead, living ray parenchyma cells may recognize signals of *Poria cocos* colonization and may have activated the phenylpropanoid biosynthesis and flavonoid biosynthesis pathways, producing polyphenols and flavonoids to defend against fungal invasion [45].

### 4.5. Selective Accumulation of Mineral Elements by Poria cocos Reflecting Preferential Utilization

In the analysis of mineral elements, the contents of Ca and K were high in pine wood before colonization, while the contents of Ca, Mg, and K were elevated after colonization. In *Poria cocos* sclerotia, P and K showed relatively high contents, indicating that fungal colonization was associated with the release of Ca and Mg, which were not subsequently incorporated into the fungal sclerotia. The high P content in *Poria cocos* sclerotia reflects a possible preferential accumulation of *Poria cocos* for phosphorus, which is consistent with the nutrient acquisition strategy of saprophytic fungi that preferentially uptake elements involved in energy metabolism and nucleic acid synthesis [46]. The K content remained relatively high in pine wood before and after colonization as well as in *Poria cocos* sclerotia, with no significant differences among the three groups, suggesting that K exists in a freely available form in wood, with high bioavailability but limited fungal utilization capacity [47]. In summary, *Poria cocos* exhibits a clear preference for mineral element utilization in pine wood, specifically absorbing elements related to energy metabolism and nucleic acid synthesis, which reflects a nutrient acquisition strategy characteristic of resource exploitation in wood substrates.

### 4.6. Limitations of This Study

This study integrated DIA quantitative proteomics and nutritional analysis to explore molecular and nutritional shifts in pine wood upon *Poria cocos* colonization. Several aspects could be improved in future research. First, host and fungal materials cannot be separated experimentally, so the precise origin of upregulated proteins cannot be fully clarified from proteomic data alone. Second, the proposed mechanistic insights are derived from correlative omics and nutritional data; further functional experiments are helpful to validate these hypotheses, such as verifying protease secretion by *Poria cocos*. Third, the association between proteomic profiles and nutritional indicators is mainly based on qualitative trend comparison. Subsequent quantitative correlation analysis would strengthen the integration of omics and phenotypic data. Moreover, only static samples before and after colonization were examined. Dynamic sampling across the colonization process may provide more comprehensive information on fungus–wood interaction.

## 5. Conclusions

This study demonstrated substantial proteomic and nutritional changes in pine wood associated with colonization by *Poria cocos*. In this work, uncolonized pine wood and pine wood after *Poria cocos* colonization were used as research materials, and DIA quantitative proteomics and nutritional composition analysis were conducted to investigate changes in protein expression and nutrient composition before and after *Poria cocos* colonization of pine wood. By searching against protein sequences from an in-house *Pinus massoniana* database, a total of 2396 proteins were quantified. A total of 784 DEPs were identified, including 595 significantly downregulated proteins and 189 significantly upregulated proteins. The downregulated proteins were derived mainly from structural proteins of pine wood degraded by *Poria cocos.* The upregulated proteins may originate from defense responses of living cells in pine wood. Since fungal mycelia were present in the colonized wood, fungal and host proteins could not be experimentally separated. Therefore, the upregulated proteins may also originate from the metabolic activity of *Poria cocos* mycelia or a combination of both; their exact origin and function require further experimental validation.GO enrichment analysis revealed that proteins with significant changes before and after *Poria cocos* colonization were associated mainly with protein degradation processes, indicating extensive degradation of pine wood proteins during colonization.KEGG analysis indicated that defense mechanisms in pine wood were activated, specifically reflected by the activation of phenylpropanoid biosynthesis and flavonoid biosynthesis pathways. By analyzing the changes in the nutritional components of pine wood and *Poria cocos* sclerotia before and after colonization, it was found that total sugars and soluble proteins decreased significantly, respectively. These findings are consistent with the hypothesis that *Poria cocos* mainly utilizes total sugars and soluble proteins in pine wood as nutrient sources for growth. In contrast, total polyphenols and total flavonoids increased by 640.38% and 687.09%, respectively, as a possible consequence of putative defense responses triggered in living pine wood cells. Mg, P, Ca, K, total nitrogen, total free amino acids, and crude fat showed no significant changes before or after colonization (*p* > 0.05). The decrease in soluble protein was consistent with the significant enrichment of protein degradation processes in GO analysis, indicating that *Poria cocos* colonization is associated with reduced abundance of pine wood proteins. The decrease in total sugars was consistent with the 595 downregulated proteins and pathways related to carbohydrate metabolism, indicating that *Poria cocos* colonization involves extensive utilization of carbohydrates in pine wood. The increases in total polyphenols and total flavonoids after colonization were consistent with phenylpropanoid biosynthesis and flavonoid biosynthesis pathways among the 189 upregulated proteins, indicating that *Poria cocos* colonization activates defense mechanisms in living cells of pine wood. In this study, a protein sequence database for *Pinus massoniana* was constructed, providing a data foundation for proteomics-related research on this species. By combining DIA quantitative proteomics with nutritional composition analysis, we provided evidence supporting putative mechanisms by which *Poria cocos* colonization of pine wood induces protein degradation and defense responses were elucidated. It was further demonstrated that during colonization, *Poria cocos* extensively utilizes total sugars and soluble proteins in pine wood.

In summary, this study characterizes proteomic and nutritional changes in *Pinus massoniana* wood during *Poria cocos* colonization. Combined analyses provide correlative evidence for substrate utilization and host defense, enriching omics resources of *P. massoniana* and offering insights into the obligate saprophytic traits of *Poria cocos*. The proposed mechanistic inferences are derived from omics correlations and require further functional validation. These findings lay a foundation for understanding the nutrient requirements of *Poria cocos*, support the development of alternative cultivation substrates, and the proteomic data facilitate the targeted design of artificial substrates matching the nutritional characteristics of this fungus.

## Figures and Tables

**Table 1:** Summary of identification results.

Database	Peptides	Identified Proteins	Quantification Proteins
140,347	4298	2444	2396

**Table 2:** Statistics of differential expression analysis results.

Statistical Index	Number	Percentage (Relative to All Quantified Proteins)
Total identified proteins	2444	——
Total quantified proteins	2396	100.00%
Total DEPs	784	32.72%
Upregulation	189	7.89%
Downregulation	595	24.83%

**Table 3:** Changes in nutrient components of pine wood before and after *Poria cocos* colonization.

Nutritional Components	Group Q (Uncolonized Pine Wood)	Group H (Pine Wood Colonized by *Poria cocos*)	Change Rate	*p*-Value
Total sugar (mg/g)	307.3 ± 6.2	196.1 ± 29.3	**↓36.19%**	**<0.001**
Total polyphenols (mg/g)	3.3 ± 0.6	24.1 ± 4.3	**↑640.38%**	**<0.001**
Total flavonoids (mg/g)	2.2 ± 0.4	17.5 ± 5.1	**↑687.09%**	**<0.001**
Magnesium (g/kg)	0.34 ± 0.11	0.53 ± 0.05	**↑57.06%**	>0.05
Total nitrogen (g/kg)	2.7 ± 0.5	3.1 ± 0.1	↑14.3%	>0.05
Soluble protein (mg/g)	0.13 ± 0.05	0.05 ± 0.01	↓62.01%	**<0.05**
Total free amino acids (mg/g)	0.05 ± 0.02	0.06 ± 0.01	↑29.00%	>0.05
Phosphorus (g/kg)	0.11 ± 0.02	0.16 ± 0.04	↑45.5%	>0.05
Potassium (g/kg)	0.74 ± 0.08	1.07 ± 0.40	↑44.6%	>0.05
Calcium (g/kg)	0.93 ± 0.16	1.14 ± 0.25	↑19.4%	>0.05
Crude fat (%)	0.010 ± 0.002	0.011 ± 0.002	↑6.61%	>0.05

Table Note: Data are presented as mean ± standard deviation (SD). ↑ indicates a significant increase in the content of the nutrient component in Group H compared with Group Q; ↓ indicates a significant decrease in the content of the nutrient component in Group H compared with Group Q. *p*-value < 0.05 represents a statistically significant difference, and *p*-value < 0.001 represents a highly statistically significant difference; *p*-value > 0.05 indicates no statistically significant difference between the two groups.

**Table 4:** Integrative interpretation of variations in nutritional components and differentially expressed proteins.

Nutritional Components	Change Rate	*p* Value	Associated Proteins/Pathways	Biological Significance
Soluble protein	↓62.01%	<0.05	GO:0030163 (Protein catabolic process) Protein decomposition process	Proteases secreted by *Poria cocos* degrade pine wood proteins
Total sugar	↓36.19%	<0.001	595 downregulated proteins; glycolysis and carbohydrate metabolism pathways (ko00500, ko00010)	*Poria cocos* extensively utilizes sugars in pine wood as a carbon source for growth.
Total polyphenols	↑640.38%	<0.001	ko00940 (phenylpropanoid biosynthesis)	Activation of pine wood defense mechanisms and phenylpropanoid biosynthesis pathway
Total flavonoids	↑687.09%	<0.001	ko00941 (flavonoid biosynthesis)	Activation of pine wood defense mechanisms and flavonoid biosynthesis pathway

## Data Availability

The raw transcriptomic data presented in this study are openly available in the NCBI BioProject database under accession number PRJNA1513453 (https://www.ncbi.nlm.nih.gov/bioproject/PRJNA1513453 accessed on 30 August 2026). The raw proteomic data have been deposited in the iProX integrated proteome resource under subproject accession number IPX0018936003. These proteomic data are currently under a temporary embargo and can be accessed for peer review via the following private link: https://www.iprox.cn/page/SSV024.html;url=1786788876187WGI3 accessed on 30 August 2026, with the access code: Vqdn. The data will be made publicly available upon publication of this manuscript.

## Supplementary Materials

The following supporting information can be downloaded at: https://www.mdpi.com/article/10.3390/jof12090666/s1, Table S1: Original complete buffer formula.
