# Supplementary material for: Mechanisms of Poria cocos Wood Colonization: Host Nutrient Depletion and Secondary Metabolite Defense
Source: J Fungi (Basel). 2026 Sep 4;12(9):666. doi: 10.3390/jof12090666 (PMC13608451; doi:10.3390/jof12090666)
Supplement: Supplementary file 1 [file jof-12-00666-s001.zip › jof-4438267-supplementary.pdf]

Table S1 Original complete buffer formula:

Ingredient	Concentration
Sucrose	0.7 M
Tris	0.5 M
HCl	30 mM
DTT	50 mM
KCl	0.1 M
EDTA	2 mM
PMSF	1% (w/v)
